# Prevalence, clinical characteristics and outcomes ofvalvular atrial fibrillation in a cohort of African patientswith acute heart failure: insights from the THESUS-HFregistry

**DOI:** 10.5830/CVJA-2017-051

**Published:** 2018

**Authors:** U Sani Mahmoud, A Davison Beth, Cotter Gad, Edwards Christopher, M Mayosi Bongani, S Ogah Okechukwu, Damasceno Albertino, B Ojji Dike, Dzudie Anastase, Kouam Kouam Charles, Mondo Charles, Suliman Ahmed, Yonga Gerald, Ba Abdou, Maru Fikru, Alemayehu Bekele, Sliwa Karen

**Affiliations:** Department of Medicine, Bayero University and AminuKano Teaching Hospital, Kano, Nigeria; Momentum Research, Inc, Durham, North Carolina, UnitedStates of America; Momentum Research, Inc, Durham, North Carolina, UnitedStates of America; Momentum Research, Inc, Durham, North Carolina, UnitedStates of America; Department of Medicine, GF Jooste and Groote SchuurHospitals, University of Cape Town, Cape Town, South Africa; Department of Medicine, University College Hospital, Ibadan, Nigeria; Faculty of Medicine, Eduardo Mondlane University, Maputo, Mozambique; Department of Medicine, University of Abuja Teaching Hospital, Abuja, Nigeria; Department of Internal Medicine, Douala General Hospital and Buea Faculty of Health Sciences, Douala, Cameroon; Department of Internal Medicine, Douala General Hospital and Buea Faculty of Health Sciences, Douala, Cameroon; Uganda Heart Institute, Kampala, Uganda; Faculty of Medicine, University of Khartoum, Khartoum, Sudan; Department of Medicine, Aga Khan University, Nairobi, Kenya; Service de Cardiologie, Faculty of Medicine, Dakar, Senegal; Addis Cardiac Hospital, Addis Ababa, Ethiopia; Addis Cardiac Hospital, Addis Ababa, Ethiopia; Hatter Institute for Cardiovascular Research in Africa, Department of Medicine, Faculty of Health Sciences, University of Cape Town, South Africa

**Keywords:** valvular, atrial fibrillation, heart failure, sub-Saharan Africa

## Abstract

**Introduction:**

Rheumatic heart disease (RHD) is the commonest cause of valvular heart disease and a common cause of heart failure in sub-Saharan Africa (SSA). Atrial fibrillation (AF) complicates RHD, precipitates and worsens heart failure and cause unfavourable outcomes. We set out to describe the prevalence, clinical characteristics and outcomes of valvular atrial fibrillation in a cohort of African patients with acute heart failure (AHF).

**Methods:**

The sub-Saharan Africa Survey of Heart Failure (THESUS-HF) was a prospective, observational survey of AHF in nine countries. We collected demographic data, medical history and signs and symptoms of HF. Electrocardiograms (ECGs) were done in a standard fashion. AF was defined as either a history of AF or AF on the admission ECG. Using Cox regression models, we examined the associations of AF with all-cause death over 180 days and a composite endpoint of all-cause death or readmission over 60 days.

**Results:**

There were 1 006 patients in the registry. The mean age was 52.3 years and 50.8% were women. AF was present in 209 (20.8%) cases. Those with AF were older (57.1 vs 51.1 years), more likely to be female (57.4 vs 49.1%), had significantly lower systolic (125 vs 132 mmHg) and diastolic (81 vs 85 mmHg) blood pressure (BP), and higher heart rates (109 vs 102 bpm). Ninety-two (44%) AF patients had valvular heart disease. The presence of AF was not associated with the primary endpoints, but having valvular AF predicted death within 180 days.

**Conclusion:**

AF was present in one-fifth of African patients with AHF. Almost half of the AF patients had valvular disease (RHD) and were significantly younger and at risk of dying within six months. It is important to identify these high-risk patients and prioritise their management, especially in SSA where resources are limited.

Rheumatic heart disease (RHD) remains an important cause of heart failure in sub–Saharan Africa (SSA),^1^,^2^ and RHD associated with valvular atrial fibrillation (AF) is also common on the continent.^3^ Among the regions enrolled in the Randomised Evaluation of Long–Term Anticoagulation Therapy in Atrial Fibrillation (RE–LY–AF), a global prospective registry that enrolled patients presenting to an emergency department with AF, RHD was present in 22% of African patients compared with 2% of North American patients.^3^

The risk of AF increases multiple–fold in the presence of heart failure (HF) and valvular disease.^4^ The prognostic influence of the presence of AF in HF remains controversial, with some studies illustrating an independent adverse effect on mortality rate.^5^,^6^ In a recent meta–analysis of 16 studies comprising more than 50 000 patients with chronic HF, Mamas and colleagues showed that AF was associated with an adverse effect on total mortality rate.^7^

From a cohort of 1 006 African patients admitted with acute heart failure (AHF) and enrolled in the sub–Saharan Africa Survey of Heart Failure (THESUS–HF) registry, we analysed the burden, clinical characteristics and outcomes of AF in general and valvular AF in particular among acute HF patients in sub–Saharan Africa.

## Methods

THESUS–HF was a prospective, multicentre, international observational survey of acute HF in 12 cardiology centres from nine countries in sub–Saharan Africa.^2^ All participating centres had a physician trained in clinical cardiology and echocardiography.

Patients, who were older than 12 years, admitted with dyspnoea as the main complaint, and diagnosed with acute HF based on symptoms and signs that were confirmed by echocardiography (de novo or decompensation of previously diagnosed HF), were enrolled consecutively. Patients excluded were those with acute ST–elevation myocardial infarction, severe known renal failure (patients undergoing dialysis or with a creatinine level of > 4 mg/dl), nephrotic syndrome, hepatic failure or another cause of hypoalbuminaemia.

Written informed consent was obtained from each subject who was enrolled into the study. Ethical approval was obtained from the ethical review boards of the participating institutions, and the study conformed to the principles outlined in the Declaration of Helsinki.

Details of data collection have been previously described.^2^ In brief, we collected demographic data, detailed medical history, vital signs (blood pressure, heart rate, respiratory rate and temperature), and signs and symptoms of HF (oxygen saturation, intensity of oedema and rales, body weight and levels of orthopnoea). Assessments were done at admission and on days one, two and seven (or discharge if earlier).

Electrocardiograms were done and read using standard reference ranges. All ECGs were read centrally at the Momentum Research Inc by one cardiologist and reviewed by a second cardiologist. ECGs were analysed for conduction or rhythm disturbances, evidence of myocardial ischaemia/infarction or hypertrophy. AF was defined as either a history of documented AF or a finding of AF on the admission ECG. The information obtained was entered in the database registry together with other clinical data.

A detailed echocardiographic assessment of ventricular contractility, valvular structure and function, as well as regional wall abnormalities was performed. All echocardiographic procedures were undertaken by trained physicians, and measurements were made according to the American Society of Echocardiography Guidelines.^8^ The probable primary cause of HF was provided by the investigators, based on the European Society of Cardiology (ESC) guidelines 9 as recently applied in the chronic HF cohort of the Heart of Soweto Study.^10^ RHD was defined based on clinical and echocardiographic criteria.^11^,^12^

Information on readmissions and death, with respective reasons and cause, was collected over the six–month follow up. Outcomes of interest were readmission or death within 60 days, and death within 180 days.

Laboratory evaluations provided by the local institutions and intravenous and oral medications were recorded at admission, and on days one, two and seven (or discharge if earlier). Vital signs (blood pressure, heart rate, respiratory rate and temperature), and signs and symptoms of HF (including oxygen saturation, intensity of oedema and rales, body weight, levels of orthopnoea) were assessed at the same time points. Changes in dyspnoea and well–being relative to admission were assessed on days one, two and seven (or discharge if earlier).

Subjects who were discharged after admission were evaluated at one and six months’ follow up. At these time points, patients were evaluated for signs and symptoms of HF, laboratory evaluations were performed, and oral medications recorded. Readmissions and death, with respective reasons and cause, over the six months of follow up were collected.

## Statistical analyses

All data were processed at the central coordinating centre at Momentum Research, Durham, North Carolina, USA. Data were analysed with the use of SAS version 9.3 (SAS Institute, Cary, North Carolina). Summary statistics (mean, SD, median, and 25th and 75th percentiles) are provided for continuous variables and frequencies for categorical variables. Unless otherwise stated, a chi–squared test was used for categorical variables, the Cochran– Mantel–Haenszel test for ordinal variables, and Wilcoxon test for continuous variables to examine comparisons between groups.

AF classification was based on subjects either having a history of AF or the presence of AF on an ECG taken at admission. Baseline characteristics by AF status are presented, as well as characteristics between patients with valvular and non–valvular AF. Comparisons between valvular and non–valvular AF patients are presented to examine differences in the following outcomes: length of index hospitalisation, time to first rehospitalisation within 60 days, all–cause mortality within 180 days, and the composite endpoint of time to all–cause mortality or rehospitalisation within 60 days.

For length of index hospitalisation, least–square means and the difference between least–square means are presented. For time–to–event outcomes, Cox regression models were used with times for patients without the event of interest being censored at the earlier of the last date the patient was known to be alive or the period of interest for the specific outcome. Kaplan–Meier estimates, hazard ratios and 95% confidence intervals are given, with the log–rank test used for comparison between groups.

Two patients who were classified with AF but were missing valvular disease status were excluded from tables comparing valvular disease in only those patients with AF. Imputation for missing values was done only when examining associations after adjustment for potential confounders using multivariable models. For multivariable modelling, these two patients were counted as having neither valvular AF nor non–valvular AF. Anticoagulation use over time is also presented as the frequency of use by AF and by valvular/non–valvular disease in only those patients with AF by time point.

The prognostic value of valvular and non–valvular AF was examined in multivariable models for the outcomes all–cause mortality within 180 days and the composite endpoint all–cause mortality or rehospitalisation within 60 days. The multivariable models were adjusted for significant clinical covariates from multivariable prognostic models previously constructed for these outcomes in the overall THESUS–HF registry.^13^

To account for missing data, multiple imputations were used with seven imputed datasets. Of the 11 baseline variables included in the multivariable model for 180–day mortality, 278 (27.6%) patients were missing one variable, 57 (5.7%) were missing two, nine (0.9%) were missing three, and four (0.4%) were missing four or more. Of the seven baseline variables included in the multivariable model for 60–day death or HF rehospitalisation, 205 (20.4%) patients were missing one variable, 44 (4.4%) were missing two, five (0.5%) were missing three, and none was missing four or more. Rubin’s algorithm was used for averaging parameter estimates across the imputed data–sets.^14^,^15^

## Results

There was a total of 1 006 patients in the THESUS–HF registry. The mean (SD) age of the patients was 52.3 (18.3) years, 511 (50.8%) were women, and the predominant race was black African (98.5%). As reported previously,^16^ the primary aetiology of heart failure was most commonly hypertension (n = 363, 39.5%), followed by idiopathic dilated cardiomyopathy (n = 136, 14.8%) and rheumatic valvular heart disease (n = 137, 14.9%), with ischaemic HF in only 72 (7.8%) patients.

AF was present in 209 (20.8%) of the 1 006 patients. In the previous THESUS–HF publication,^2^ prevalence was documented to be 18.3% because only those who had AF on the admission ECG were analysed. Table 1 shows the baseline patient characteristics by AF status. In both the AF and non–AF groups, about 80% of the patients were in NYHA class II or III one month prior to admission. Compared to the patients without AF, the patients with AF were older (mean age 57.1 vs 51.1 years) and more likely to be female (57.4 vs 49.1%). They also had significantly lower systolic (125 vs 132 mmHg) and diastolic (81 vs 85 mmHg) blood pressures and higher mean heart rates (109 vs 102 bpm).

**Table 1:** Baseline patient clinical characteristics by atrial fibrillation status

*Variables*	*Atrial fibrillation^1^ (n = 209)*	*No atrial fibrillation^1^ (n = 797)*	*p-value^2^*
Age (years)	57.1 (17.73), 60.0 (46.0–70.0)	51.1 (18.26), 52.0 (36.0–65.0)	< 0.0001
Gender: female, n (%)	120 (57.4)	391 (49.1)	0.0328
Black African, n (%)	203 (97.6)	781 (98.7)	0.2291
BMI (kg/m^2^)	24.94 (5.712), 24.73 (21.02–28.08)	24.85 (5.836), 23.88 (20.83–27.99)	0.4736
SBP (mmHg)	124.5 (29.86), 120.0 (102.0–145.0)	131.9 (34.27), 130.0 (108.0–150.0)	0.0128
DBP (mmHg)	80.6 (19.54), 80.0 (67.0–90.0)	85.3 (21.19), 82.0 (70.0–100.0)	0.0032
Heart rate (bpm)	109.3 (28.02), 108.0 (90.0–124.0)	102.2 (19.29), 103.0 (90.0–114.0)	0.0021
History of hypertension, n (%)	110 (52.9)	446 (56.2)	0.3959
Hyperlipidaemia, n (%)	9 (4.5)	81 (10.4)	0.0109
Stroke, n (%)	7 (3.4)	18 (2.3)	0.3613
Ischaemic heart disease, n (%)	11 (5.3)	71 (8.9)	0.0849
Valvular disease, n (%)	92 (44.4)	180 (22.7)	< 0.0001
Peripheral vascular disease, n (%)	3 (1.4)	9 (1.1)	0.7072
Anaemia, n (%)	99 (49.0)	390 (51.0)	0.6183
Pericardial disease, n (%)	9 (4.3)	44 (5.6)	0.4815
Cardiomyopathy, n (%)	80 (38.8)	336 (42.6)	0.3243
LVEF (%)	42.31 (15.721), 41.90 (31.00–52.00)	38.74 (16.623), 37.00 (25.40–50.00)	0.0022
LVEF < 40%, n (%)	82 (41.6)	405 (55.3)	0.0155
eGFR (ml/min/1.73 m^2^)	78.257 (40.114), 70.782 (49.422–98.271)	84.685 (49.838), 77.735 (55.929–104.20)	0.0522
Renal dysfunction, n (%)	10 (5.0)	63 (8.4)	0.1020

^1^Mean (SD), median (first quartile – third quartile) for a continuous variable and frequency (per cent) for a categorical variable.

^2^Chi-squared test for a categorical variable, CMH for an ordinal variable and Wilcoxon test for a continuous variable.

BMI, body mass index; SBP, systolic blood pressure; DBP, diastolic blood pressure; LVEF, left ventricular ejection fraction; eGFR, estimated glomerular filtration rate.

Ninety–two (44%) of the 207 AF patients had valvular heart disease. Compared with those without valvular disease, these patients were younger (mean age 52 vs 61 years), had lower systolic blood pressure (120 vs 128 mmHg) and higher left ventricular ejection fraction (LVEF) (47 vs 38%). Fifty–seven per cent had LVEF ≥ 45%. Among patients with non–valvular AF, 61% had hypertensive heart disease. The other baseline characteristics were similar in the two groups (Table 2).

**Table 2:** Baseline patient clinical characteristics versus valvular and non-valvular AF

*Variables*	*Valvular AF^1^ (n = 92)*	*Non-valvular AF1 (n = 115)*	*p-value^2^*
Age (years)	52.2 (18.98), 52.0 (38.5–65.5)	60.8 (15.74), 64.0 (53.0–72.0)	0.0005
Gender: female, n (%)	59 (64.1)	60 (52.2)	0.0838
Black African, n (%)	91 (98.9)	111 (97.4)	0.4245
BMI (kg/m^2^)	24.89 (6.482), 24.76 (20.91–27.91)	24.90 (5.005), 24.52 (21.23–28.11)	0.7187
SBP (mmHg)	119.9 (24.39), 112.0 (100.0–133.0)	127.9 (33.38), 124.5 (108.0–150.0)	0.0699
DBP (mmHg)	78.4 (17.01), 79.0 (65.0–90.0)	82.2 (21.29), 80.0 (68.0–94.0)	0.2521
Heart rate	111.7 (29.68), 109.0 (92.0–127.0)	107.4 (26.86), 107.0 (90.0–120.0)	0.4319
History of hypertension, n (%)	38 (41.8)	70 (60.9)	0.0064
Hyperlipidaemia, n (%)	4 (4.6)	4 (3.6)	0.7244
Stroke, n (%)	3 (3.3)	3 (2.6)	0.7706
Ischaemic heart disease, n (%)	4 (4.3)	6 (5.2)	0.7719
Valvular disease, n (%)	92 (100.0)	0 (0.0)	< 0.0001
Peripheral vascular disease, n (%)	2 (2.2)	1 (0.9)	0.4235
Anaemia, n (%)	46 (51.7)	51 (45.9)	0.4196
Pericardial disease, n (%)	3 (3.3)	6 (5.2)	0.5030
Cardiomyopathy, n (%)	29 (32.2)	50 (43.5)	0.1003
LVEF (%)	47.21 (14.440), 46.00 (40.00–58.00)	38.20 (15.506), 36.50 (27.00–45.70)	< 0.0001
LVEF < 40%, n (%)	22 (24.7)	59 (55.7)	< 0.0001
eGFR (ml/min/1.73 m^2^)	78.731 (39.738), 74.123 (48.772–92.796)	77.676 (40.914), 69.039 (49.709–100.88)	0.7990
Renal dysfunction, n (%)	4 (4.3)	6 (5.6)	0.6961

^1^Mean (SD), median (first quartile – third quartile) for a continuous variable and frequency (per cent) for a categorical variable.

^1^Chi-squared test for a categorical variable, CMH for an ordinal variable and Wilcoxon test for a continuous variable.

BMI, body mass index; SBP, systolic blood pressure; DBP, diastolic blood pressure; LVEF, left ventricular ejection fraction; eGFR, estimated glomerular filtration rate.

Anticoagulation prescription rates were low in this cohort of patients and decreased progressively over time. At six months, only 22% of patients with AF were on oral anticoagulants. For the AF patients, 33% of the patients with valvular AF and 12% of those with non–valvular AF were on anticoagulants at six months’ follow up (Table 3). For aspirin, a greater proportion of patients with AF than without AF were on aspirin one month prior to admission (29 vs 20%), but on and after admission the proportions did not differ significantly.

**Table 3:** Anticoagulation and aspirin use versus time

*Parameters*	*1 month prior to admission*	*Admission*	*Day 30*	*6 months*
Atrial fibrillation (n = 209)				
Anticoagulation, n (%)	16/136 (11.8)	107/205 (52.2)	31/116 (26.7)	22/101 (21.8)
Aspirin, n (%)	39/135 (28.9)	68/203 (33.5)	67/115 (58.3)	60/102 (58.8)
No atrial fibrillation (n = 797)				
Anticoagulation, n (%)	19/444 ( 4.3)	234/786 (29.8)	66/499 (13.3)	22/326 ( 6.8)
Aspirin, n (%)	88/443 (19.9)	283/784 (36.1)	303/499 (60.7)	189/327 (57.8)
p-value^1^	0.0013	< 0.0001	0.0003	< 0.0001
p-value^2^	0.0266	0.4905	0.6269	0.8546
Atrial fibrillation				
Valvular (n = 92)				
Anticoagulation, n (%)	12/63 (19.0)	48/91 (52.8)	20/34 (58.8)	15/45 (33.3)
Aspirin, n (%)	20/63 (31.8)	33/90 (36.7)	13/33 (39.4)	28/46 (60.9)
Non-valvular (n = 115)				
Anticoagulation, n (%)	4/71 ( 5.6)	58/112 (51.8)	10/81 (12.4)	7/56 (12.5)
Aspirin, n (%)	18/70 (25.7)	34/111 (30.6)	54/81 (66.7)	32/56 (57.1)
p-value^1^	0.0168	0.8915	< 0.0001	0.0117
p-value^2^	0.4420	0.3667	0.0073	0.7035

^1^Chi-squared test for comparison of anticoagulation use.

^2^Chi-squared test for comparison of aspirin use.

As shown in Table 4, the mean length of the hospital stay was 1.6 days longer in patients with valvular AF than for patients with non–valvular AF, although this was not statistically significant (p = 0.14). Patients were followed for a median of 180 days. Of 151 total deaths over 180 days, 20 occurred among patients with valvular AF and 13 among those with non–valvular AF.

**Table 4:** Outcomes versus valvular disease status in patients with atrial fibrillation

Variable	Valvular atrial fibrillation (n = 92)	Non-valvular atrial fibrillation (n = 115)	Effect (95% CI3)	p-value
Length of index hospitalisation^1^	11.2	9.6	1.63 (–0.56–3.83)	0.1438
Rehospitalisation within 60 days^2^	6.0	11.3	0.48 (0.15–1.52)	0.2046
Death within 180 days^2^	24.8	13.2	2.11 (1.05–4.24)	0.0320
Death or rehospitalisation within 60 days^2^	19.1	15.5	1.32 (0.66–2.65)	0.4268

^1^LS means: difference presented for length of index hospitalisation.

^2^Kaplan–Meier event rate.

^3^Hazard ratio from Cox regression model presented for rehospitalisation, death, and death/rehospitalisation outcome.

A total of 70 patients, among them two with valvular AF and 10 with non–valvular AF, were lost to follow up within six months but were included in estimated rates as censored observations. Four patients with valvular AF and 11 with non–valvular AF were rehospitalised within 60 days; 16 patients in each group were either rehospitalised or had died by day 60. The unadjusted risk of 180–day mortality rate in patients with valvular AF was twice that in patients with non–valvular AF [hazard ratio (HR) 2.11, 95% CI: 1.05–4.24, p = 0.032], while the unadjusted risks of 60–day rehospitalisation did not differ significantly. Without adjustment for potential confounding factors, neither valvular nor non–valvular AF was associated with 60–day readmission, while valvular but not non–valvular AF was associated with 180–day all–cause mortality (Table 5).

**Table 5:** Associations of valvular and non-valvular atrial fibrillation with all-cause death within 180 days

		Unadjusted hazard ratio		Multivariable adjusted hazard ratio	
Variable	HR for a change of	(95% CI1)	p-value	(95% CI1)	p-value
Valvular atrial Fibrillation	Yes vs no	1.61 (1.00–2.58)	0.0475	1.61 (0.99–2.62)	0.0563
Non-valvular atrial fibrillation	Yes vs no	0.69 (0.39–1.21)	0.1949	0.70 (0.39–1.26)	0.2331
Male gender	Yes vs no	–	–	1.36 (0.96–1.92)	0.0859
Haemoglobin (g/dl)	1 unit Increase	–	–	0.93 (0.87–1.00)	0.0551
HIV positive	Yes vs no	–	–	1.82 (1.08–3.06)	0.0239
Current or former smoker	Yes vs no	–	–	0.49 (0.24–0.99)	0.0479
Malignancy	Yes vs no	–	–	3.05 (1.24–7.54)	0.0157
Hx of cor Pulmonale	Yes vs no	–	–	2.04 (1.26–3.30)	0.0038
SBP (mmHg)	10 units Increase	–	–	0.85 (0.80–0.90)	< 0.0001
Orthopnoea	(2/3 vs 0/1)	–	–	2.32 (1.06–5.10)	0.0360
Peripheral Oedema	(2/3 vs 0/1)	–	–	1.76 (1.15–2.69)	0.0094
Rales	(2/3 vs 0/1)	–	–	1.71 (1.11–2.63)	0.0155
Creatinine (mg/dl)2	1.55 vs 0.90	–	–	1.37 (1.06–1.77)	0.0239

^1^Hazard ratio from Cox regression model. ^2^Appropriate transformation used due to the non-linear relationship between predictor and outcome.

**Fig. 1:**
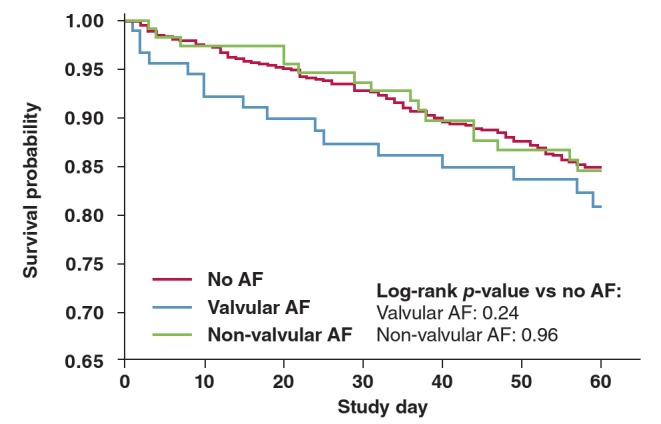
Kaplan–Meir plot: death or rehospitalisation up to day60 based on presence and type of AF.

**Fig. 2:**
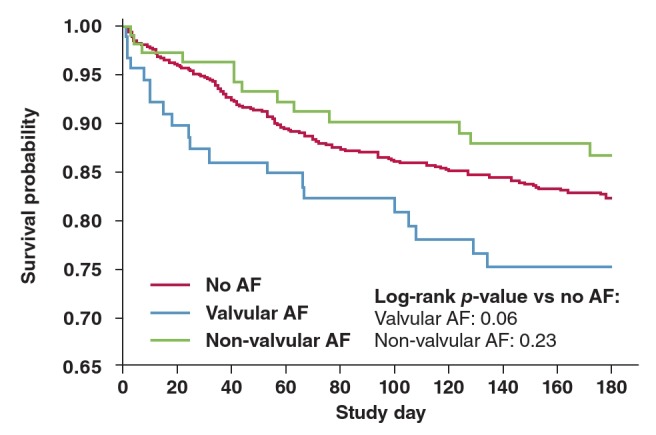
Kaplan–Meir plot: death up to day 180 based on presenceand type of AF.

Adjustment for clinical characteristics found to be prognostic of the outcomes^13^ and therefore possible confounders had little effect on these estimated associations. Valvular AF was not a significant predictor of all–cause death or readmission within 60 days (Fig. 1) (HR 1.39, 95% CI: 0.80–2.42, p = 0.24) but was associated with all–cause death within 180 days (HR 1.61, 95% CI: 0.99–2.62, p = 0.056) (Fig. 2). On the other hand, non–valvular AF was not a significant predictor of either all–cause death or readmission within 60 days (HR 0.99, 95% CI: 0.58–1.68, p = 0.96) (Fig. 1) or the outcome all–cause death within 180 days (HR 0.70, 95% CI: 0.39–1.26, p = 0.23) (Fig. 2).

## Discussion

We found AF to be present in 20.8% of AHF patients. Fortyfour per cent of the patients with AF had valvular heart disease. Sixty–one per cent of the patients with non–valvular AF had hypertension. The presence of AF was not associated with the primary endpoints, but having valvular AF predicted death within 180 days.

To the best of our knowledge, this is the first sub–Saharan African study to assess the prevalence and clinical characteristics of AF in patients hospitalised for AHF. Significantly, this study describes an important sub–population of patients with AF, namely AF due to valvular (mostly rheumatic) heart disease, which is still relatively prevalent in sub–Saharan Africa.^17^–^21^

The prevalence of AF was 21%, which is generally lower than that reported in previous studies, ranging between 23 and 41%.^22^–^25^ Among acutely decompensated HF patients, 20 to 35% will be in AF at presentation.^26^ This difference may be due to the fact that in sub–Saharan Africa, patients with AHF are younger and have less incidence of ischaemic heart disease. Indeed in the Heart of Soweto HF cohort, AF occurred in only 6.6% of all 2 393 HF cases within the entire study cohort.^17^ This however was not in the acute decompensated HF setting.

RHD was the third commonest cause of HF in the THESUS–HF study after hypertension and idiopathic dilated cardiomyopathy.^2^ Almost half of all those with AF in this study had valvular disease compared to 23% in those without AF. Valvular heart disease has long been associated with the development of AF. Populationbased estimates from the Framingham data revealed that valvular disease was associated with a 1.8–fold increase of risk of AF in men and a 3.4–fold increased risk in women.^27^

Although any valvular pathology can be related to AF, stenotic left–sided valvular lesions (and in particular RHD) have the highest prevalence rates. Severity of the obstruction follows a dose–response relationship: prevalence of AF was 9.1% in patients with mild–to–moderate aortic stenosis and 33.7% among those with severe stenosis.^28^,^29^ Likewise, the prevalence of AF varies with the complexity of RHD: from 16% with isolated mitral regurgitation to 29% with isolated mitral stenosis, to 52% with coexisting mitral regurgitation and stenosis, and to 70% with mixed mitral and tricuspid valve disease.^30^

We found low rates of anticoagulation in this cohort. In a prospective study of AF patients in Cameroon, only 34% of patients with an indication for oral anticoagulation received it;31 similar to the 33% of patients with AF who received warfarin in the Heart of Soweto study.^17^ In this study 52% of our patients with AF received oral anticoagulants. By contrast, a much higher percentage of patients received an anticoagulant in Senegal, where in a retrospective hospital–based study, anticoagulation with warfarin was established in 62% of cases.^32^ In the REMEDY registry,^18^ 40.7% of patients had indications for oral anticoagulants and they were prescribed in 69.5% of patients. The use of oral anticoagulants was high in patients with mechanical heart valves (91.6%) and AF (68.6%), but low in those with mitral stenosis in sinus rhythm with either dilated left atrium or left atrial thrombus (20.3%).

A study at a private urban referral teaching hospital in Nairobi, Kenya, found that 80% of patients with AF and a CHADS2 score of 2 received anticoagulation.^33^ Similarly, a recent observational multicentre national registry in South Africa indicated that 75% of patients with AF were on warfarin for stroke prevention.^34^ We did not collect data on the quality of anticoagulation control in this study.

The presence of AF was not associated with all–cause death or readmission within 60 days, but having valvular AF predicted death within 180 days. Eapen and colleagues^35^ investigated the associations between AF and early outcomes of patients with HF. They found AF to be associated with 30–day mortality in patients with preserved ejection fraction but not in those with reduced ejection fraction.

Our data should be interpreted in the context of their limitations. The variable timing of the ECG may have affected the specific results obtained, given that the ECGs were accepted if they were recorded within two weeks of admission. Secondly, our results are drawn from a population of young AHF patients predominantly with systolic dysfunction. Consequently, these findings may not apply to older patients or to those with preserved LVEF. Finally, the study was conducted in selected specialised centres, and only patients who consented to the study were enrolled; therefore not all patients admitted with AHF were represented and the study’s generalisability may be limited. However, we have increased our understanding of the growing importance of cardiovascular disease in this population, who now suffer from the double burden of communicable and non–communicable diseases.

## Conclusion

AF is present in one–fifth of sub–Saharan African patients with AHF. Almost half of the AF patients have valvular disease (RHD) and are significantly younger. Valvular AF was associated with all–cause death within 180 days but was not a significant predictor of all–cause death or readmission within 60 days. The prescription rates of anticoagulation with warfarin were low in this cohort.
